# Effects of prenatal exposure factors on birth outcomes through mediation of favorable fetal growth conditions using structural equation modeling

**DOI:** 10.1371/journal.pone.0249664

**Published:** 2021-04-27

**Authors:** Aweke A. Mitku, Temesgen Zewotir, Delia North, Prakash Jeena, Rajen N. Naidoo

**Affiliations:** 1 School of Mathematics, Statistics and Computer Science, College of Agriculture Engineering and Science, University of KwaZulu-Natal, Durban, South Africa; 2 Department of Statistics, College of Science, Bahir Dar University, Bahir Dar, Ethiopia; 3 Discipline of Paediatrics and Child Health, School of Clinical Medicine, College of Health Sciences, University of KwaZulu-Natal, Durban, South Africa; 4 Discipline of Occupational and Environmental Health, School of Nursing and Public Health, College of Health Sciences, University of KwaZulu-Natal, Durban, South Africa; University of Mississippi Medical Center, UNITED STATES

## Abstract

**Background:**

Birth weight, birth length, and gestational age are major indicators of newborn health. Several prenatal exposure factors influence the fetal environment. The aim of the study was to investigate the effect of prenatal exposure factors, including socio-demographic, behavioural, dietary, physical activity, clinical and environmental on birth outcomes through the mediation of Favourable Fetal Growth Conditions (FFGC).

**Methods:**

Data was obtained from six hundred and fifty-six Mother and Child in the Environment birth cohort study in Durban, South Africa from 2013 to 2017. We adopted structural equation models which evaluate the direct and indirect effects by allowing multiple simultaneous equations to incorporate confounding and mediation.

**Results:**

A significant direct and indirect effect of FFGC on newborn weight, length, and gestational age was seen. Gestational weight gain and maternal body mass index in the first trimester exerted a mediation effect between maternal behavioural risk factors and FFGC. Similarly, the level of physical activity during pregnancy was associated with decreased gestational weight gain. The effects of maternal characteristics on newborn weight, length, and gestational age were largely indirect, operating through FFGC as a latent variable.

**Conclusions:**

Gestational weight gain and maternal pre-gestational BMI were observed to mediate the association between prenatal behavioural risk factors and favourable fetal growth conditions.

**Trial registration:**

Retrospectively registered from 01 March 2013.

## Background

Fetal development is a global public health concern because growth in utero is a good indicator of perinatal and postnatal health [1]. Poor fetal growth during pregnancy is an important risk factor for stillbirth, neonatal death, and morbidity [2]. It is also a predictor of adverse health outcomes in later life, including cardiovascular and metabolic disease [3]. Many fetuses delivered with a lower than expected birth weight are small because their growth in utero has been impaired [4]. Low birthweight and length, or shortened gestational age are indicators of an abnormal fetal environment and can be indications of adverse health outcomes [5]. Recent studies showed that adverse birth outcomes could be related to unfavourable fetal growth conditions, which may have long-term health consequences for outcomes such as cardiovascular disease [6–8], blood pressure [9, 10] and risk of diabetes [11] in adolescence and adult life.

Studies have examined the relationship between prenatal characteristics and birth outcomes with the mediation effect of favourable fetal growth conditions (FFGC) [5, 12, 13]. However, methodological issues seem a challenge to permeate these claims to validate with data. Recent studies in Filipino islands [5] and the United States [13] employed a structural equation model. They demonstrated the existence of a latent variable, called Favorable Fetal Growth Conditions, constructed with indicator variables of the three birth outcomes birth weight, birth length and gestational age. It is the understanding behind the fetal origins hypothesis [14].

FFGC is associated with dimensions that play an essential role in prenatal development such as environmental, genetic or epigenetic factors [12]. Due to the difficulty of directly measuring the fetal environment, studies often rely on birthweight as a proxy for fetal conditions, assuming rather than testing the plausibility of FFGC latent variable. The use of a single observed measure as a proxy variable has shortcomings [15], it may also result in misclassification of infants as growth-restricted. It also assumes that birthweight is a perfectly reliable indicator of fetal conditions, thus ignoring any possible measurement error [13]. Fetal conditions measures have also included birth length [16], and gestational age [17]. The latent variable approach properly accounts for measurement error in each observed indicator. It also allows measuring both the direct and indirect effect of maternal characteristics. Bollen et al. confirmed that the model with the FFGC latent variable better fits the data than a model with only a direct effect of maternal characteristics [5].

A previous study [18] in Durban city have reported Oxides of Nitrogen (NOx) as a key pollutant, and it was found that increased levels of ambient air pollution to be a major health concern for mothers as well as fetal growth [19]. This is the first African study that investigated the mediation effects of FFGC between maternal and environmental characteristics and birth outcomes. Consequently, rather than focusing on only individual-level explanations of maternal prenatal exposure, it is important also to adjust factors that may be beyond their control, such as prenatal air pollution. Thus, this study adopted structural equation models to investigate the effect of prenatal exposure characteristics on birth outcomes through the mediation of Favourable Fetal Growth Conditions.

## Methods

### Data and variables

The Mother and Child in the Environment (MACE) birth cohort is a study with ongoing recruitment in Durban, South Africa. The study enrolled a cohort of 996 pregnant women from March 2013 to May 2017 from public sector antenatal clinics in the study areas. From a total of 996 women, 687 of them were enrolled subjects followed up during their pregnancy, through to labour and delivery, with the remainder having had multiple pregnancies (n = 2); miscarriages (n = 55); still births (n = 25); and termination of pregnancy (n = 2) and were therefore excluded from the cohort. A further 225 participants relocated outside the areas of interest. Excluding 31 participants with postdates birth (gestational age above 42 weeks) reduced the effective sample size to 656 mother-child pairs. This study was developed with data from MACE birth cohort in Durban, South Africa in the period of 2013 to 2017. The inclusion, exclusion criteria of selection of participants is as follows. All pregnant women that were at a gestational age of fewer than 20 weeks and resident for the full duration of the pregnancy in the geographical area within which the clinic was located as well as for the follow-up period of 5–6 years in the cohort were recruited in the study. Women with multiple pregnancies were excluded from the study.

### Maternal prenatal exposure factors

This study considered both observed and latent variables as prenatal exposure factors. Observed variables include environmental (exposure to NOx pollution), clinical (gestational weight gain (GWG), pre-gestational body mass index (BMIT1), and HIV status (HIV Positive)), maternal age (Mage), and parity (multiparous). Latent variables: diet (eight dietary patterns derived from factor analysis detailed in our previous study [20]), physical activity (walking and physical exercise, PhyEx), behavioural (alcohol, smoking (Smoker), and exposure to tobacco smoke (PSmoker)), Maternal low socio-economic status (LSES) (employment (unemployment), maternal income (Low income (Linc)), education (primary or less (PLEduc)), and housing (Low socio-economic housing (LSEH)) (Table 1). GWG was obtained as the difference in kilograms between the weight measured within 24 to 36 weeks of third trimester and within the first 16 weeks of first trimester, and maternal BMI (in kg/m^2^) was calculated using first-trimester weight and height.

**Table 1:** The birth outcome measures summary statistics by prenatal exposure factors.

Factors	N	Mean birth length (SD)	Mean birthweight (SD)	Mean gestational age (SD)
**Maternal smoking (Smoker)**				
**Yes**	42	49.1(3.7)	2892.7(543.7)	37.9(3.0)
**No**	614	49.4(4.3)	3069.1(490.1)	38.7(2.0)
**Tobacco smoke exposure (PSmoker)**				
**Yes**	211	49.4(4.2)	3038.8(507.0)	38.7(2.3)
**No**	445	49.4(4.3)	3066.8(489.8)	38.7(1.9)
**Alcohol consumption**				
**Yes**	50	48.9(4.0)	2967.4(525.7)	38.6(2.2)
**No**	606	49.4(4.3)	3065.3(492.3)	38.7(2.0)
**Primary or less maternal education (PLEduc)**				
**Yes**	15	48.3(6.1)	3001.8(647.4)	37.8(2.2)
**No**	641	49.4(4.2)	3059.1(491.6)	38.7(2.0)
**Maternal Unemployment (Unemp)**				
**Yes**	120	49.1(4.2)	3053.4(493.5)	38.7(1.9)
**No**	536	49.44(4.2)	3058.8(504.8)	38.7(2.1)
**Low maternal annual income (LInc)**				
**Yes**	576	49.4(4.3)	3052.6(497.6)	38.7(2.1)
**No**	80	49.1(4.0)	3095.6(478.5)	38.9(1.7)
**Low socio-economic housing (LSEH)**				
**Yes**	208	49.3(4.4)	3022.2(521.2)	38.6(1.9)
**No**	448	49.4(4.2)	3074.4(482.3)	38.7(2.1)
**Multiparous**				
**Yes**	126	49.3(4.8)	3101.1(496.3)	38.5(2.5)
**No**	530	49.4(4.1)	3047.5(494.8)	38.7(1.9)
**Child gender**				
**Female**	323	49.0(4.4)	3046.6(482.6)	38.7(2.1)
**Male**	333	49.7(4.1)	3068.7(507.5)	38.7(2.0)
**HIV status**				
**Positive**	212	49.3(4.1)	3047.1(511.6)	38.6(2.0)
**Negative**	444	49.4(4.3)	3062.9(487.6)	38.7(2.1)
**Median exposure level of NOx (μg/m^3^)**	656	34.4 (8.5)		
**Maternal age (years)**	656	26 (5.7)		

LSEH Flat, terraced flat, apartment building or Informal housing; Informal dwelling is a makeshift structure not erected according to approved architectural plans, for example, shacks or shanties in informal settlements or in backyards [28].

Low maternal annual income (Less than US$2000).

Prenatal NOx exposure (*μg*/*m*^3^) for participants in the MACE birth cohort was predicted, using land use regression model based on pre-selected geographic predictors, such as land use types, road length, topography, population and housing density with the methodology used in the European Birth Cohorts (ESCAPE). The parameter estimates were used to predict NOx exposure at the residential addresses of the study participants, as detailed in Muttoo et al. [21, 22]. Written, informed consent was obtained from the mother for their participating children and for their own consent. Follow-ups of the study were approved by the respective ethics committees at the University of KwaZulu-Natal Medical School.

### Birth outcomes

Three birth outcomes were birthweight (BW) in grams, length (BL) in centimetres and gestational age (GA) in weeks measured from the last menstrual period date. BW and BL were measured by nurses at birth delivery using project scales and custom length boards respectively. All three birth outcomes were retained as continuous variables. As some birth outcomes are gender-differentiated infant gender (Female)) was added as a confounder.

### Structural equation model

**Fig 1:**
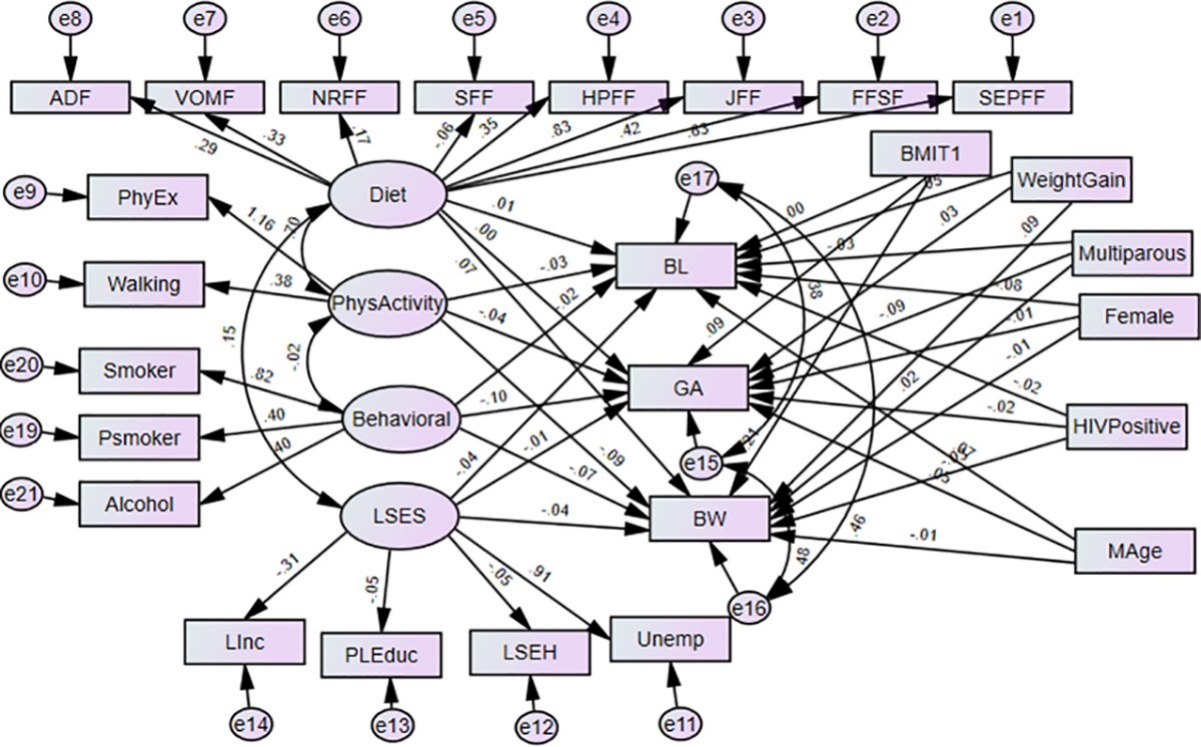
Structural equation model of direct effects of prenatal exposure characteristics on infant’s birth outcomes. BW-Birthweight; BL-Birth length; GA- Gestational age; NOx- Oxides of Nitrogen; GWG- gestational weight gain; BMIT1- pre-gestational body mass index in the first trimester; and Mage- maternal age. Latent variables: dietary factors (EFSF- energy foods and snacks; FFSF-spreads and fast foods; JFF- junk foods factor; PRFF-protein-rich foods; SFF- starch foods; NRFF-nuts and rice foods; VRFF-vegetable-rich foods; and ADF-alcoholic drinks); PhyEx- physical exercise; PSmoker- passive smoker; LSES -low socio-economic status, Linc- Low income; PLEduc- primary or less education; LSEH-Low socio-economic housing; Unemp- unemployed.

**Fig 2:**
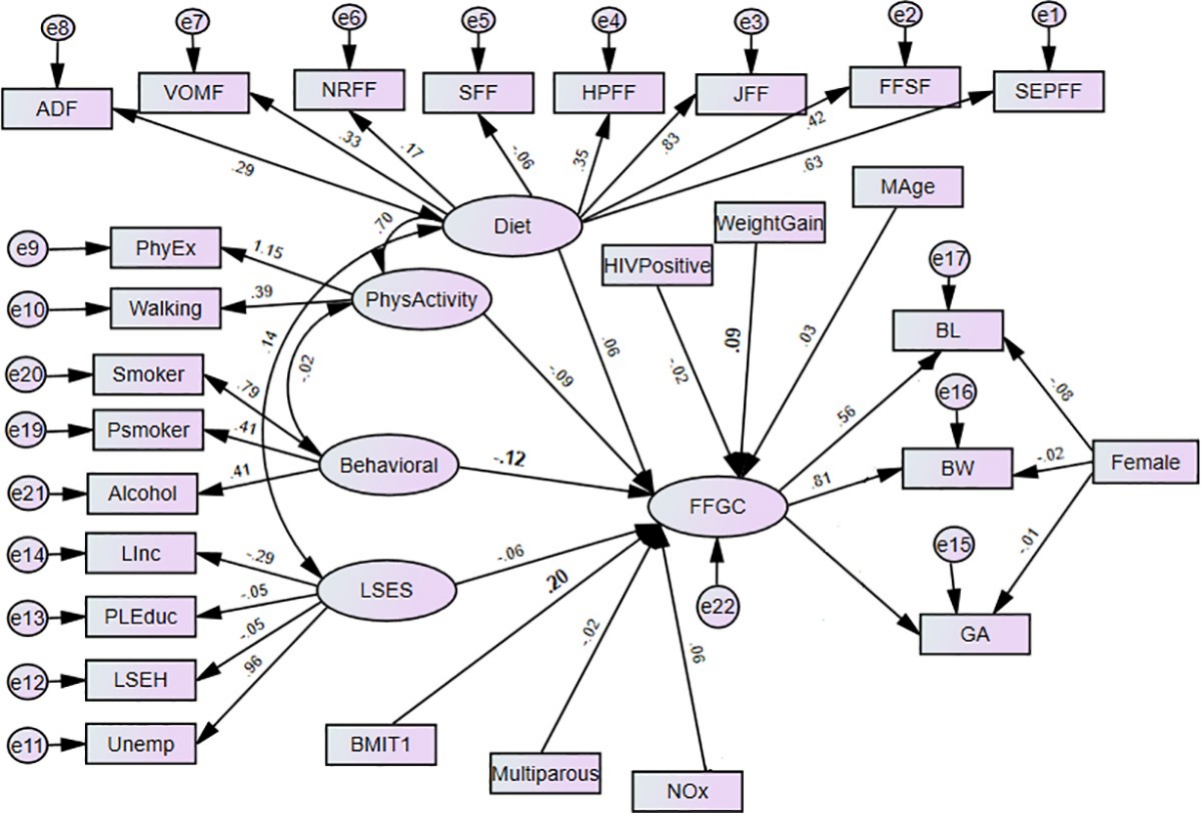
The effects of prenatal exposure characteristics on birth outcomes (BW, BL, GA) with FFGC as a mediating latent variable (Model 2) BW-Birthweight; BL-Birth Length; GA- Gestational Age; NOx- Oxides of Nitrogen; GWG- Gestational Weight Gain; BMIT1- Pre-gestational body mass index; and Mage- maternal age. Latent variables: dietary factors (EFSF- energy foods and snacks; FFSF-spreads and fast foods; JFF- junk foods factor; PRFF-protein-rich foods; SFF- starch foods; NRFF-nuts and rice foods; VRFF-vegetable-rich foods; and ADF-alcoholic drinks); PhyEx- physical exercise; PSmoker- passive smoker; LSES -low socio-economic status, Linc- Low income; PLEduc- primary or less education; LSEH-Low socio-economic housing; Unemp- unemployed.

**Fig 3:**
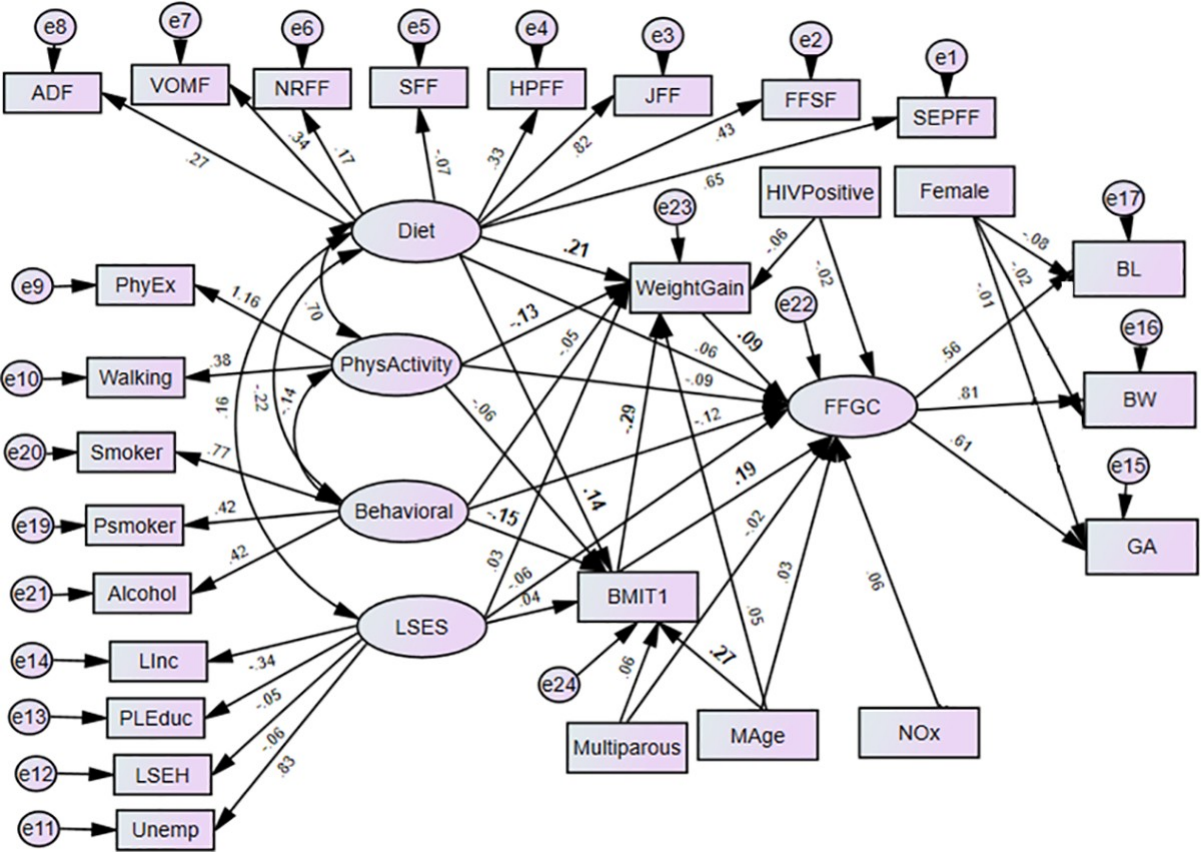
Prenatal maternal exposure characteristics on birth outcomes (BW, BL, GA) through FFGC, gestational weight gain and pre-gestational BMI (Model 3) BW-Birthweight; BL-Birth Length; GA- Gestational Age; NOx- Oxides of Nitrogen; GWG- Gestational Weight Gain; BMIT1- Pre-gestational BMI; and Mage- maternal age. Latent variables: dietary factors (EFSF- energy foods and snacks; FFSF-spreads and fast foods; JFF- junk foods factor; PRFF-protein-rich foods; SFF- starch foods; NRFF-nuts and rice foods; VRFF-vegetable-rich foods; and ADF-alcoholic drinks); PhyEx- physical exercise; PSmoker- passive smoker; LSES -low socio-economic status, Linc- Low income; PLEduc- primary or less education; LSEH-Low socio-economic housing; Unemp- unemployed.

The structural equation models (SEMs) are multivariate statistical models that involve relationships among endogenous and exogenous latent variables, accounting for measurement error. It provides a general framework for modelling stochastic dependence that arises through cause-effect relationships between random variables by minimizing the effect of residual confounding in associations, especially in observational studies [23]. It allows mediating effect inclusion on the exposure and outcome variables. Latent variables are unobserved factors that summarize the information and reduce the dimension of observed variables. In path diagrams of SEM, Figs 1–3, the ovals signify latent variables and observed variables are shown in rectangles. SEMs are employed to analyze our data.

We developed a measurement model in which birthweight, birth length, and gestational age are together treated as indicators of a latent factor variable that we call “favourable fetal growth conditions (FFGC)”. We construct factor scores for latent FFGC to compare the performance of the individual components of fetal conditions with the more general measure. Factor scores are predicted values for an underlying latent variable using the factor score regression method [24]. The regression method is by far the most common method for estimating factor scores. It maximizes the correlation between the factor scores and the latent variable they reflect. In our analysis, these correlations are quite high.

### Mediation models

Mediation between prenatal characteristics and birth outcomes (BW, BL, and GA) via latent variable FFGC and gestational weight gain was assessed in a single SEM. The mediation model seeks to discover and explicate the underlying mechanism of an observed relationship existing between a dependent and an independent variable through including a third explanatory variable, which is typically known as a mediator variable [25]. We have employed the causal steps approach for the assessment and evaluation of the mediation model [26]. One of our interests is whether BW, BL, and GA are distinct birth outcomes with distinct causes or if it is reasonable to view them as having a common dependence on a latent FFGC variable. The latter suggests a collection of factors coalesce into a variable that simultaneously influences what happens to BW, BL, and GA. A model without FFGC represents the fact that there is no mediating, common latent variable for BW, BL, and GA and that each variable has a separate and distinct set of relationships to maternal prenatal exposure. Furthermore, maternal BMI and gestational weight gain were examined as mediators of the relationships between the prenatal exposure factors and birth outcomes through FFGC. We represent these ideas in three models that we compare.

In Model 1 both the latent and observed maternal prenatal characteristics have direct effects on GA, BW, and BL without a mediating effect (see Fig 1). In this model, the observed endogenous variables are BW, BL, and GA. Each endogenous variable has an error represented by a short arrow that points toward it. The errors or disturbances contain all of the other influences on the measure or latent variables not included in the model. In addition, we allow the disturbances or errors in these latter variables to correlate with each other.

Adopted from Bollen [27], Model 2 (see Fig 2) uses the FFGC latent variable to mediate the effects of the maternal characteristics on BW, BL, and GA. In Model 2, we hypothesize a single latent variable, FFGC that constituted an unobserved measure of a composite of favourable or unfavourable conditions for fetal growth, which simultaneously affect BW, BL and GA. The exogenous variables viz. socio-economic, behavioural, dietary, physical activity and environmental (i.e., exposure to NOx) factors might have both favourable or unfavourable impact fetal growth. In this model, if there is improvement in FFGC, we expect that BW, BL, and GA each will improve; if FFGC declines, we expect each to reduce. Child gender (Female) does not have a direct effect on FFGC, as it constituted an intrinsic characteristic of the infant, while other variables characterize the environment for fetal growth. NOx is also allowed to directly affect BW as a significant relationship was seen in our previous research [22] and BL as well.

In Model 3, we further extended Model 2 for possible mediation effects of an observed variables gestational weight gain and pre-gestational BMI on birth outcomes through FFGC. To assign a scale to the latent variable, we fixed the path between the latent variable FFGC and one outcome, the birthweight, to 1 (the standard deviation of the BW variable being the highest). In Models 2 and 3 there is no necessity for these correlated errors of the birth outcomes suggest that these correlations are due to a common dependence on FFGC. In all of the models, the error variables are assumed uncorrelated with all exogenous variables. In addition, we assume that all measurement errors for the indicators of BW, BL, and GA are uncorrelated with each other. All exogenous variables are allowed to correlate with each other. The models were estimated by using the robust maximum likelihood approach; specifically, an expectation-maximization algorithm. Data analyses were performed in SPSS AMOS version 25.0. For the analyses, a p-value< 0.05 is considered statistically significant.

## Results

The descriptive summary statistics for our data is given in Table 1. The overall mean birthweight of the babies in the cohort was 3106·5 grams (g) (SD: 538.2g). The mean maternal age was 26 years (SD: 5.7 years). The overall median level of NOx was 34.4 μg/m^3^ (range 3–45 μg/m^3^).

The path diagrams of the fitted models are shown in Figs 2 and 3. The single-headed arrows indicate causal effects and the associated parameter values show the standardized estimates. Covariances between errors were considered in the model fit and reduced for the sake of complexity of the SEM diagram. The chi-square of the models was statistically significant, which is expected as the sample size is moderately large (N = 656). Nonetheless, other fit indices indicated a good fit of the model. The model fit diagnostics results showed that the fitted models 2 and 3 had CMIN/DF ratios below 5. The goodness of fit measure RMSEAs was all below the acceptable value 0.08 except model 1. Hence, Model 3 showed that BMI and gestational weight gain act a mediating role between the prenatal exposure characteristics and birth outcomes through the latent variable FFGC. Model 3 was found to have small AIC and was selected as the best model (Table 2). The graphic representations Figs 2 and 3 shows the standardized estimates of model 2 and model 3 respectively with significance indicated through bolding. In model 2, the latent variable behavioural maternal habits had statistically significant negative effects on FFGC (β = -0.12, p-value< = 0.037). The pre-gestational BMI (β = 0.20, p-value< 0.01) and gestational weight gain (β = 0.09, p-value = 0.020) were significantly and positively associated with FFGC (Fig 2).

**Table 2:** Goodness of fit measures for structural equation models.

	CMIN	DF	PCMIN/DF	RMSEA	AIC
**Model 1**	1829.3	299	6.1	.088	1931.0
**Model 2**	1405.7	298	4.7	.075	1619.7
**Model 3**	1267.0	288	4.4	.072	1511.5

AIC: Akaike Information Criteria

Table 3 displays total, direct and indirect effects of prenatal exposure characteristics on FFGC and gestational weight gain for Model 3. Model 3 shows that the direct negative effect of behavioral latent variable on FFGC was not significant (β = -0.12, p-value = 0.05), but it had a negative and significant indirect relationships via gestational weight gain (β = -0.03, p-value< 001) (Fig 3 and Table 3). Moreover, the total effect is significant, which indicates that gestational weight gain fully mediated the association between behavioural latent variable and FFGC. The direct effect of pre-gestational BMI on FFGC was positive and significant (β = 0.19, p-value< 0.01), but the indirect effect via gestational weight gain was negative (-0.03, p-value = 0.020). When considering the total effect of pre-gestational BMI (direct effect and via gestational weight gain), the effect was stronger (β = 0.17, p-value< 0.01). This implies gestational weight gain showed partial mediation on the relationship between maternal pre-gestational BMI and FFGC. Physical activity latent variable (β = -0.02, p-value = .001) showed a significant indirect effect on FFGC via gestational weight gain. The total effect of physical activity was found borderline significant (β = -0.11, p = 0.051). Mediation of the association between physical activity during pregnancy and FFGC via gestational weight gain was not achieved. The latent variable diet (β = 0.04, p-value< 0.01) and maternal age (β = 0.05, p-value< 0.01) only showed a positive indirect effect on FFGC via gestational weight gain. The total effect of these variables on FFGC did not achieve statistical significance (Fig 3 and Table 3).

**Table 3:** Maximum likelihood ratio bootstrapped estimates of direct, indirect and total effects under Model 3.

Independent variables	Outcome variables					
	FFGC via GWG			Gestational weight gain via BMI		
	Direct effect	Indirect effect	Total effect	Direct effect	Indirect effect	Total effect
**GWG**	0.092*	NA	0.092*	NA	NA	NA
**Diet**	0.057	0.041**	0.098	0.201**	-0.038*	0.163*
**Physical activity**	-0.089	-0.022*	-0.112	-0.128**	0.017	-0.111*
**Pre-gestational BMI**	0.193**	-0.026*	0.168**	-0.287**	NA	-0.287**
**Behavioural**	-0.116	-0.030**	-0.146*	-0.051	0.042**	-0.009
**Maternal age**	0.030	0.045**	-0.075	0.054	-0.074**	-0.074*

** = p-value <0.01

* = p-value <0.05

On the other hand, there were partial mediator effects of pre-gestational BMI due to the latent variable maternal diet on gestational weight gain. Maternal diet significantly predicted gestational weight gain (β = 0.20, p-value< 0.01). Diet also had a significant indirect effect on gestational weight gain via BMI (β = -0.04, p-value = 0.03). Maternal pre-gestational BMI had a negative effect on gestational weight gain (β = -0.29, p-value< .001). Furthermore, a full mediating effect of maternal pre-gestational BMI was observed between maternal age and gestational weight gain (Fig 3). Firstly, maternal age significantly affected BMI (β = .27, p-value< 0.01). Secondly, BMI significantly affected gestational weight gain (β = - 0.29, p-value< .001). Moreover, the effect of maternal age on gestational weight gain was insignificant. Thus, BMI plays a role in mediating the relationship between maternal age and gestational weight gain (Fig 3). The latent variable behavioral had only significant indirect effect on gestational weight gain (β = 0.04, p-value< 0.01). No significant effect was observed between prenatal exposure to NOx and birth outcomes via FFGC.

## Discussion

A structural equation model utilised to model the multidimensional response outcome variables namely BW, BL, and GA into a single latent variable allowed for the composite comparison of birth outcomes, with FFGC. This model was fitted with bootstrapped bias-corrected standard errors and confidence intervals.

Our study was able to consider for several important prenatal exposure factors during pregnancy: demographic, socio-economic, behavioural, diet, physical activity, and medical as well as maternal exposure to NOx as an environmental factor in data from MACE birth cohort. All these factors are separately known to reduce gestational age, birthweight and birth length, representing different dimensions of fetal growth, which might be captured in the favourable/unfavourable fetal growth conditions latent variable [12]. The mediation effect of pre-gestational BMI and gestational weight gain on birth outcomes through FFGC was an important finding. Mother’s pre-gestational BMI and gestational weight gain were positively associated with birth outcomes through FFGC, and BMI was found to be the strongest predictor of FFGC.

Previous studies in infants from the Philippines [5], US [13], and French [12], which described the mediation effects of FFGC on birth outcomes, have recommended replication of studies with FFGC latent variable in different populations and settings. Similarly, our study found that SEM with mediating FFGC fits the data better than a model without such a mediating effect. Likewise, we found that gestational weight gain and pre-gestational BMI mediated the effect of prenatal behavioural risk factors on birth outcomes through FFGC. This implies that infants from mothers with risk behaviours (smoker, exposed to environmental tobacco smoke and consume alcohol) were linked to unfavourable fetal growth conditions. This is consistent with similar studies which found smoking during pregnancy have significant negative effects on FFGC [5, 13]. In addition to maternal smoking, our study advanced it by including exposure to environmental tobacco smoke and alcohol intake were accounted for in our behavioural latent variable.

Studies that consider the only birthweight as fetal growth found positive associations of higher maternal pre-gestational BMI and GWG with the risk of larger birthweight of infants [29–31]. Our study showed a higher pre-gestational BMI has been associated with lower gestational weight gain. Consistent with our finding, a study in France reported that pregnancy weight gain is negatively related to pre-pregnancy BMI [32]. Our study further highlighted that pre-gestational BMI played a mediator role on the effects of maternal diet on gestational weight gain. In other words, maternal pre-gestational BMI has had a mediating effect on the relationship between maternal diet and gestational weight gain. We have also found that increased level of physical activity during pregnancy, physical exercises as well as walking, was associated with decreased GWG. Some studies, including randomized, controlled trials, have also demonstrated the inverse relationship between physical activity and GWG, supporting the role of institute of medicine recommendations that prenatal care providers offer counselling on physical activity [33, 34].

The strength of this study is the use of the latent variable approach which allows three commonly available birth outcomes BW, BL, and GA as a proxy for the fetal environment instead of using only birthweight on data from a developing country. This provides a very general and convenient framework for statistical analysis that includes several multivariate procedures such as regression analysis, discriminant analysis, and canonical correlation, as special cases. Another strength of our study is the use of maternal exposure risk factors of adverse birth outcomes, such as BMI, exposure to NOx, current and passive smoking habits, alcohol consumption and physical activity during pregnancy. Even “well-behaved” random errors can bias the coefficient estimates of the health effects of not only the fetal environment indicators (BW, BL, and GA) but also of the other control variables that are part of the analysis. This happens even if the control variables are free of error [35]. For this reason, we estimated structural equation models that attempt to minimize measurement error. These findings suggest that analytic approaches used are important extensions of the usual methods, but furthermore, there are important public health implications for exposed communities, already marginalized or socio-economically vulnerable.

The main limitation of this study is the use of a single average pollutant NOx exposure levels during the whole pregnancy. The effect of exposure to NOx at different trimesters may have a differential effect on birth outcomes. Another limitation of the study is the absence of the weekly or monthly weight gain measurements. This may have influenced the association with the latent variable and needs to be investigated in other areas with repeated measurement of gestational weight gain.

## Conclusions

Structural equation modeling allowed an investigation of maternal pre-gestational BMI and gestational weight gain as mediators of prenatal exposures and birth outcomes via FFGC. This methodological approach takes into account the correlation between multiple birth outcomes. Consequently, prenatal behavioural risk exposure was related to severe birth outcomes. Among others, maternal smoking, environmental exposure to smoking and alcohol consumption during pregnancy led to unfavourable fetal growth conditions. Furthermore, high level of mother’s physical exercise was found in the reduction of mothers’ gestational weight gain.

## List of abbreviations

BMI: Body Mass Index
BW: Birthweight
BL: Birth Length
FFGC: Favourable Fetal Growth Conditions
GA: Gestational Age
GWG: Gestational Weight Gain
MACE: Mother and Child in the Environment
NOx: Oxides of Nitrogen
SEM: Structural Equation Models
